# Effects of somatic treatments on suicidal ideation and completed suicides

**DOI:** 10.1002/brb3.2381

**Published:** 2021-10-17

**Authors:** Elise M Hawkins, William Coryell, Stephen Leung, Sagar V. Parikh, Cody Weston, Paul Nestadt, John I. Nurnberger, Adam Kaplin, Anupama Kumar, Ali A. Farooqui, Rif S. El‐Mallakh, For the National Network of Depression Centers Suicide Prevention Task Group

**Affiliations:** ^1^ Department of Psychiatry and Behavioral Sciences University of Louisville Louisville Kentucky USA; ^2^ Roy J. and Lucille A. Carver College of Medicine The University of Iowa Iowa City Iowa USA; ^3^ Department of Psychiatry University of Michigan Ann Arbor Michigan USA; ^4^ Department of Psychiatry and Behavioral Sciences Johns Hopkins University School of Medicine Baltimore Maryland USA; ^5^ Department of Psychiatry Indiana University School of Medicine Indianapolis Indiana USA

**Keywords:** antidepressants, clozapine, esketamine, ketamine, lithium, protective, suicide

## Abstract

**Objective:**

This work was undertaken to define and characterize the role of currently available somatic treatments in psychiatry in either increasing or reducing the risk for suicide.

**Methods:**

Members of the Suicide Prevention Task Group of the National Network of Depression Centers performed a literature review of somatic treatments known to increase or reduce the risk for suicide. The reviews ventured to include all relevant information about the risk for both suicide ideation and completed suicides.

**Results:**

Lithium and clozapine are the only two somatic treatments that have high‐quality data documenting their antisuicide effects in mood disorders and schizophrenia, respectively. Lithium discontinuation is also associated with increased suicide risk. Ketamine and esketamine may have a small, but immediate, antisuicide effect. Despite the recent Food and Drug Administration approval of esketamine use in depressed suicidal patients, the small disproportional overrepresentation of suicide in subjects who had received esketamine versus placebo (3 vs. 0 among > 3500 subjects) requires ongoing evaluation. The purported antisuicide effect of electroconvulsive therapy is based on low‐quality data. The effect of antidepressants is not at all clear. There appears to be direct evidence for antidepressants increasing suicidal ideation and the risk for suicide over the short‐term in young people, but indirect (low quality) evidence that antidepressants reduce suicide risk over the long term.

**Conclusions:**

Clinicians have an expanding pharmacopeia to address suicide potential in their patients. Some of the agents with documented antisuicide effects may also increase suicidality under specific circumstances.

## Abstract 2

Systemic review of controlled trials of effect of medications on suicide revealed that lithium and clozapine have a documented antisuicide effect in bipolar illness and schizophrenia, respectively. Antidepressants appear to be associated with an increase of suicide ideation for young people acutely, but there is weak evidence that they may reduce suicide ideation or completed suicide over the long term. Ketamine, and possibly esketamine, appear to have a transient antisuicide effect.

## 1 INTRODUCTION

Suicide is a major public health concern with 800,000 people die by suicide annually, and even more attempt it (GBD 2017 Causes of Death Collaborators, 2018). Suicide is now the 10th leading cause of death in the United States, increasing by 30% since 1999 (Centers for Disease Control & Prevention, 2007), and appears to continue to be increasing particularly among veterans and youths (Goldstein et al., 2019; Hoffmire et al., 2015; Smith et al., 2019; Steelesmith et al., 2019). Globally, suicide ranks as the 18th leading cause of death across the lifespan, the second cause of death among those aged 15–29 years, and occurs at a startling rate of one person every 40 s (World Health Organization, 2020). Not only is suicide a costly burden in terms of human life, but also monetarily, accruing an estimated $ 58.4 billion annually in lost wages and medical expenditures combined in the United States (Shepard et al., 2016). In conjunction with those who have a completed suicide, an additional estimated 9.3 million adults in the United States have had thoughts of suicide within the past year, indicating the increasing need for an effective treatment for those experiencing acute or chronic suicidal ideation (Centers for Disease Control & Prevention, 2007).

Earlier research has focused on identifying risk factors for completed suicides, suicide attempts, and suicidal ideation (Tucker et al., 2015). Around 90% of suicide victims had a mental health diagnosis prior to death (De Berardis et al., 2018). Suicidal ideation is one of the major drivers of inpatient psychiatric hospitalization as a way of ensuring safety (Way & Banks, 2001; Ziegenbein et al., 2006). Nonetheless, suicides remain to be common soon after hospital discharge (Chung et al., 2017; Olfson et al., 2016). A multitude of protocols has been proposed as important for ensuring safety and reducing suicide risk, with varying success (D'Anci et al., 2019). Unfortunately, the recent increase in completed suicides (Goldstein et al., 2019; Hoffmire et al., 2015; Smith et al., 2019; Steelesmith et al., 2019) is evidence that we still have a long way to go.

Although depression is only one of the causes that leads to suicide, it is a major contributing factor in approximately half of completed suicides (Bachmann, 2018). For that reason, there is a particular focus on somatic interventions for depression as an indirect treatment for suicide ideation and prevention of suicide (D'Anci et al., 2019).

Currently, suicidal ideation is treated through somatic interventions, such as medications and/or electroconvulsive therapy (ECT) (Fink et al., 2014), and psychosocial interventions, such as psychotherapy, most specifically cognitive behavioral therapy (CBT) (D'Anci et al., 2019). Pharmacologically, there is the potential for both positive and negative effects on suicides. The effects antidepressants are controversial, but there is an association for more immediate increased risk for suicide with indirect evidence and a reduced risk over the lifetime (Brent, 2016). Medications such as lithium and clozapine have shown efficacy in lowering suicide bipolar patients and schizophrenia patients, respectively (D'Anci et al., 2019). However, lithium discontinuation may increase the risk for suicide (Baldessarini et al., 1999). Newer studies highlight the potential for ketamine or its enantiomer, esketamine, in the management of treatment‐resistant depression (TRD) and suicidal thoughts (De Berardis et al., 2018; Wilkinson et al., 2018), but with possible withdrawal depression (Lin et al., 2016) with associated increased risk for suicide (Huetteman, 2019).

The current review is a product of a subcommittee of the Suicide Prevention Taskforce of the National Network of Depression Centers with the intent to summarize the direct effect of medications on significant suicide ideation and completion. The group focused on randomized trials of specific treatments and avoided secondary or nonspecific or secondary effects such as might be seen with prednisone, ethanol, or other drugs (Icick et al., 2017; Judd et al., 2014; Larkin et al., 2017).

## 2 METHODS

Individual investigators or small subgroups undertook the review of specific agents or groups of agents. These assignments were based on research interest and background knowledge of each investigator in the group. Literature searches varied slightly, but all utilized guidelines of the Preferred Reporting Items for Systematic Reviews and Meta‐Analyses (PRISMA; http://www.prisma‐statement.org).

A literature search was performed in the electronic database, PubMed. The following phrases were used: “SSRIs and suicide,” “antidepressants and suicide,” “suicide and lithium,” “suicide and clozapine,” “suicide and ketamine,” and “suicide and esketamine” with the applied filters of: human subjects, adult population, available in English, and full‐text format. Each subgroup utilized their own criteria in choosing the relevant articles. Additional articles were found by researchers as they investigated their topic. The switch of format of PubMed in May of 2020 may have affected the results in an unclear way.

A PubMed search of “oral ketamine and suicide” yielded 24 results, of which only nine were relevant to oral ketamine, and most of these were theoretical.

For intravenous (IV) ketamine, a literature search was performed in the electronic database PubMed through July 1, 2020. The following phrase was used: “suicide and ketamine” with the applied filters of: human subjects, adult population, available in English, and full‐text format. These combined searches yielded 100 articles that were then narrowed down to 37 based on relevance to the topic. From there, only articles that looked at explicit markers of suicidality were included with a greater emphasis on evidence levels I–III. Reference lists and appendices from identified articles were also manually searched for pertinent references. These articles included six open‐label studies, 12 randomized controlled trials (RCTs), two meta‐analyses/systematic reviews, and three post‐hoc analyses. Articles were excluded if not specific to suicidal ideation and/or attempts, if the article lacked relevance to the administration of the specific medications examined in this review, if the focus of the article was simply to examine suicide in a specific population, or if the full article was not available. Literature was not excluded based on desired outcomes to limit any research bias. In addition to these electronic databases, the CDC and the WHO websites were accessed for statistical information, and the American Psychiatric Association (APA) was referenced as expert opinion for practice guidelines and recommendations on the proper treatment of suicidal patients.

Additional searches may have been performed that are not listed here. For example, “meta‐analysis suicide antidepressant” was performed in addition to “antidepressants and suicide” to double check the reference lists. These additional searches were not monitored across the entire group of researchers.

Articles were excluded if not specific to suicidal ideation and/or attempts, if the article lacked relevance to the administration of the specific medications examined in this review, if the focus of the article was simply to examine suicide in a specific population, or if suicide was a single case report or a sequence of cases not part of a randomized trial.

The quality of reviewed research was rated according to the Grading of Recommendations Assessment, Development, and Evaluation (GRADE) system, randomized trials were considered high quality if there was no obvious bias, and moderate quality if there was a risk of bias, or low quality if the data derived from observational data (Guyatt et al., 2011). These decisions were reviewed by two other coauthors (the coauthor of that particular section and RSE, who was responsible for reviewing all sections).

## 3 RESULTS

These combined searches yielded 2258 articles that were then evaluated by each subgroup investigating a particular agent. For each category, the following were obtained: SSRIs and suicide 636; lithium and suicide 306 papers that were reduced to 39 after manual review; clozapine and suicide 198; ketamine and suicide 138; and esketamine and suicide 4.

## 4 DISCUSSION

There is a significant literature regarding suicide and various treatments, but the overall quality of studies is suboptimal. There are only two somatic treatments that have been shown to reduce suicide risk in randomized, controlled trials: lithium and clozapine. Only recently has a medication, esketamine, been approved for use in depressed patients with acute suicidal ideation, but it has not been approved *for* suicidal ideation. Clinicians need to understand the role of their treatments in the prevention of this fatal outcome of psychiatric disease. Additionally, clinicians need to reach beyond the lore and preconceived notions and base their actions on the highest quality evidence available. To achieve this, the National Network of Depression Centers established a subcommittee of the Suicide Prevention Taskforce to clarify the role of somatic treatments.

### 4.1 Antidepressants

Approximately 12.7% of the U.S. population aged 12 years and over have taken an antidepressant in a single month during 2017, making this class of medications one of the three most commonly used in the United States (Pratt et al., 2017). Antidepressants are classified according to their primary mechanism of action, including serotonin reuptake inhibitors, serotonin norepinephrine inhibitors (SNRIs), monoamine inhibitors (MAOIs), tricyclics (TCAs), and others. Nearly all of the currently available agents work on the monoamines: serotonin (5HT), norepinephrine (NE), dopamine (DA), melatonin, and histamine. Serotonin‐reuptake inhibitors (SRIs, commonly and erroneously called specific serotonin reuptake inhibitors, SSRIs) are the most commonly used group of agents. With the exception of one antidepressant (bupropion), *all* currently available antidepressants will increase synaptic 5HT. This makes understanding the 5HT system very important in both mechanisms of action and suicide potential.

Despite an FDA warning regarding increased suicidal ideation in young patients, there remains a significant divide with researchers concluding that antidepressants reduce, increase, or have no effect on suicidal ideation or completed suicide(e.g., Courtet & Olié, 2014; Healy & Whitaker, 2003). For example, the rate of completed suicide among adolescents had been on a downward trend since the introduction and expanding use of SRIs in the mid‐1990s; but that trend stopped and suicide began to increase again after the FDA put out the warning regarding the increase in suicide ideation observed in young people (Bridge et al., 2008). It has been argued that these changes in suicides are related to inverse changes in antidepressant use in juveniles (Libby et al., 2007; Nemeroff et al., 2007; Olfson et al., 2008).

However, these discrepancies are the consequence of multiple issues such as terminology and definitions, analytic methods, or bias. For example, there are major differences between suicidal ideation, suicidal acts, and suicide, and their reporting has not been standardized (Meyer et al., 2010). However, standardized methods, such as the Columbia Classification Algorithm for Suicide Assessment, while more reliable (i.e., reproducible), underreport suicidal acts in pediatric populations by some 50% compared to clinician assessment (Posner et al., 2007). Thankfully, completed suicide is a rare event in medication trials, partially due to active exclusion of potentially suicidal subjects (Lorenzo‐Luaces et al., 2018), but this makes it difficult to generalize data, and to achieve statically meaningful results (Hammad et al., 2006). Additionally, there is a bias against reporting serious adverse events in the published clinical trial literature, making drugs appear safer when compared to FDA data (de Vries et al., 2016). Finally, even when using the same data sets, the method of evaluation can lead to different results, particularly when dealing with rare events (Kaminski & Bschor, 2020). Concomitant resolution of these and other problems is unlikely in the foreseeable future making it important to exercise caution for all interpretations. Nonetheless, there appear to be adequate data for some preliminary conclusions.

The FDA issued their warning after an analysis of 372 double blind randomized, placebo‐controlled trials that included nearly 100,000 adults (*n* = 99,231), which is the most comprehensive analysis available (Stone et al., 2009). The FDA used the term suicidality to encompass both suicidal ideation and suicidal acts. In the entire data set, there were eight suicides, which were more than twice as likely to occur in patients treated with antidepressants (odds ratio [OR] = 2.13, 95% confidence interval [CI] 0.41–10.99, ns due to small sample size) (Stone et al., 2009). For all patients, antidepressants were not significantly associated with any change in suicidality (OR = 0.85, CI 0.71–1.02, which is barely ns) (Stone et al., 2009). However, over a fifth (22.2%) of this sample were patients receiving antidepressants for nonpsychiatric disorders. When only the studies involving psychiatric patients (i.e., studies involving depression and anxiety) are included, antidepressants are indeed associated with a *reduced* suicidality risk (OR = 0.83, CI 0.69–1.00, *p* = .05) (Stone et al., 2009). This is probably related to the finding that suicidality is higher in people with depression than in the general population (Valuck et al., 2016).

Additionally, the FDA further described an age effect. For individuals less than 25 years old, the odds ratio for suicidal ideation (OR = 1.62, CI 0.97–2.71, ns) and suicidal acts (OR = 2.30, CI 1.04–5.09, *p* < .05) were elevated compared to placebo. This was not the case for individuals aged 25–64, where suicidal ideation was significantly reduced (OR = 0.79, CI 0.64–0.98, *p* < .05) but not suicidal acts (OR = 0.87, CI 0.58–1.29, ns). Both suicidal ideation and acts were significantly reduced in elders ≥65 years old (OR = 0.37, CI 0.18–0.76, *p* < .05; and OR = 0.06, CI 0.01–0.58, *p* < .05) (Stone et al., 2009).

Analyses that do not account for age find that suicidal *acts* may be increased in adults. One review that specifically looked at violent behaviors commented on the poor reporting in published papers, but found that in 13 out of 159 studies identified where there was sufficient data, the risk for harm or violence nearly doubled with antidepressant treatment (OR = 1.85, CI 1.11–3.08, *p* = .02) (Bielefeldt et al., 2016). An older analysis of published English language studies found a total of 21 completed suicides among 40,028 patients involved in MDD studies (0.05%) (Hammad et al., 2006). In studies performed in North America, the antidepressant‐associated increased risk was minimal or nonexistent (risk ratio = 1.07, CI 0.1–63.4, ns) (Hammad et al., 2006).

For those less than 18 years, a meta‐analysis of 22 short‐term studies submitted to European registration officials found a significant increase in suicidality (suicide ideation and suicidal acts) in subjects being treated for depression (OR = 1.67, CI 1.05–2.65, *p* < .05), but not for anxiety (OR = 1.33, CI 0.33–5.35, ns) (Wohlfarth et al., 2006). Several meta‐analyses of published studies with people younger than 19 years of age document an increase in variously defined suicidality (Dubicka et al., 2006; Hammad et al., 2006; Mosholder & Willy, 2006). For example, an analysis of 19 MDD studies with 3335 subjects found an increase in suicide related outcomes (no completed suicides, relative risk = 1.58, CI 1.02–2.45, *p* < .05) (Hetrick et al., 2012). Another analysis performed early in the millennium found that there was a significant increase in suicidal or self‐harm acts with antidepressants compared to placebo (1.70, CI 1.13–2.54, *p* = .01) (Dubicka et al., 2006). A meta‐analysis done around same time but including more trials found similar results—suicidal ideation and attempts were increased in children with antidepressant exposure by about 0.7% (CI 0.1−1.3%, *p* < .05), but the calculated number needed to harm was 143, compared to a number needed to treat of 8 (Bridge et al., 2008). Although the rarity of suicidality makes analysis more problematic, when the issue of rarity is statistically accounted for, the increase in suicidality is apparent (Julious, 2013).

A meta‐analysis of eight observational studies involving more than 200,000 patients found that SRI use was associated with an increased the risk of completed or attempted suicide among adolescents (OR = 1.92, CI 1.51–2.44, *p* < .05) (Barbui et al., 2009). Interestingly, the likelihood of self‐harm after antidepressant initiation doubled if the antidepressant dose used was higher than the modal recommended dose in younger people (<24 years, but not in people ≥ 25 years) in a matched cohort study of 162,625 community‐based Americans (hazard ratio [HR] = 2.2, CI 1.6–3.0, *p*  =  .04) (Miller et al., 2014).

Evaluations by non‐FDA authors of data of adults receiving antidepressants find a similar pattern as FDA data. For example, a mega‐analysis of adults receiving citalopram, paroxetine, sertraline, or placebo for major depression there was a significant decrease in the mean rating of suicidality on the Hamilton Rating Scale for Depression (HRSD) in subjects over 25 years old (Näslund et al., 2018). Numerous other studies also found reduction in suicidality, measured by varying rating scales, versus placebo or tricyclic antidepressants among adults taking SRIs (Beasley et al., 1991; Grunebaum et al., 2012; Gunnell et al., 2005; Hawton et al., 2015; Isacsson et al., 2009; Khan et al., 2018; Thase et al., 2017).

Duration of exposure to SRIs introduces another important variable. A meta‐analysis of 29 long‐term relapse prevention, randomized, placebo‐controlled trials including some 6934 patients found that risk with antidepressant use for completed suicide was five times higher (*p* = .102) and nine times higher for suicide attempt (*p* = .007) (Braun et al., 2016). One study accounted for the majority of suicides and suicide attempts, and exclusion of that study found that no difference is the risk for suicidal acts when patients with MDD are treated with an antidepressant or placebo (Braun et al., 2016). Similarly, a meta‐analysis of observational studies that included older MDD patients (>60 years old) found that long‐term exposure (2–11 years) was associated with an increased risk of attempting suicide (OR = 1.18, CI 1.10–1.27, *p* < .05), but not completed suicide (7–11 years, OR = 1.06, CI 0.68–1.66, ns) (KoKoAung et al., 2015). However, study design plays a role in observed outcome. In a study that enriches the rare outcome of suicide by examining completed suicides finds that 70% of subjects who died by suicide were prescribed antidepressants for at least 2 years at the time of their deaths, 90 % of which were SRIs (Castelpietra et al., 2017). In the young, if suicidality is reduced, the effect is seen for only 1 week but not beyond (1.06, CI 0.68–1.66, ns) (KoKoAung et al., 2015; Näslund et al., 2018).

There are many other variables that may play a role. For example, in a 10‐year case‐control study found that *adherence* to prescribed antidepressants was associated with a trend toward lowered suicide risk. In this study, completed suicides (cases) were compared to five controls in which all participants (cases and controls) had to be prescribed at least one antidepressant in 2 years prior to the suicide; 70% of the cases had been treated with antidepressants prior to their death (Castelpietra et al., 2017). However, only 26% of these case individuals were adherent and currently using the medication at the time of death, with a decreasing trend in suicide risk for those who were adherent to their medication regimen versus those who were not (Castelpietra et al., 2017). Alternatively, as the *severity* of the current depressive episode appears to predict better adherence, this could also be an important variable (Umetsu et al., 2015).

In a 27‐year observational study of a mixed diagnosis group, use of antidepressants was specifically related to symptom severity of symptom worsening (Leon et al., 2011). In this cohort, when severity was controlled for, the risk of a suicide attempt or an actual suicide was 20% less among patients prescribed antidepressants versus those not taking antidepressants (Leon et al., 2011).

There may some evidence that any effect on worsening of suicide may be related to serotonin. All antidepressants (SRIs, SNRI, TCAs, and MAOIs, but not bupropion) have a serotonin component. An analysis of the placebo‐controlled trials of bupropion for MDD finds 28 cases of worsening of suicidal ideation or some suicidal action among 5489 patients receiving either bupropion or placebo, a small sample size for this type of evaluation (Leon et al., 2011). Nonetheless, the OR for worsening of the suicidal ideation was 1.28 (CI, 0.59–2.86, ns), and for suicidal acts, 3.52 (CI, 0.81–24.48, ns) (Wightman et al., 2010). There were no significant differences from placebo across all age groups, but younger people (18–24 years) were more likely than older patients (>24 years) to have some increase in suicidality *independent of treatment assignment* (Wightman et al., 2010). Similar results are seen with atomoxetine, a norepinephrine reuptake inhibitor that does not interact with serotonin and has been studied predominantly for attention deficit disorders (ADDs). However, despite its lack of approval for MDD, it does get used off label for the treatment of depression (Dadashova & Silverstone, 2012). Across multiple meta‐analyses in pediatric populations, suicidality was numerically increased in atomoxetine‐treated subjects, but this was not significantly different from placebo (Bangs et al., 2008; Bangs et al., 2014; Bushe & Savill, 2013; Schwartz & Correll, 2014). Naturalistic, cohort data also fail to demonstrate increased overrepresentation of suicidality in 5–18‐year‐old youths prescribed atomoxetine (Linden et al., 2016). Similarly, in adults receiving atomoxetine, there is no difference in suicidality between atomoxetine and placebo (Bangs et al., 2014).

#### 4.1.1 Summary and potential mechanisms

Suicidal ideation or suicidal acts (collectively referred to as suicidality) are not changed or reduced in a large number of patients that receive antidepressants. Reduction in suicidality is greatest among patients with MDD with minimal evidence for an effect on suicidality in patients with anxiety disorders or nonpsychiatric indications. The antisuicide effect is greatest earlier in the course of treatment. Age is one of the major determinants of changes in suicidal risk with antidepressants. Older age is associated with a statistically significant reduction in suicidality, whereas younger age is associated with increased suicidality. The mechanism of this effect is not known, but it may be related, at least in part, to a serotonin effect, because independent evaluations of nonserotoninergic agents, bupropion and atomoxetine, do not appear to have any effect on suicidality (although this may be due to inadequate power because independent evaluation of single agents significantly reduces the sample size).

The striking difference across ages is clearly a clue to the potential mechanism of the observed suicide risk. The data point to two possible mechanisms for increased suicidality risk in young people: (1) an enrichment of subjects with a diagnosis of bipolar illness, but who have not yet declared their true diagnosis with a manic episode (Hogg et al., 2016); and (2) an overrepresentation of individuals with the genetic variant of the short form of the serotonin transporter (SERT) (Luddington et al., 2009).

Major depressive episodes frequently precede initial manic episodes in nearly half (48.6%) of prepubescent children, resulting in the misdiagnosis of bipolar depression as unipolar depression (Geller et al., 2001). Childhood adversity may also play a role so that children who present with depression in the setting of parental separation are 2.37‐fold more likely to develop bipolar disorder (BD) over the subsequent 15 years of follow‐up (Bohman et al., 2017). Adults with BD who are exposed to long‐term antidepressants can experience a destabilization of their illness with an increased number of both manic *and* depressive episodes as a consequence of antidepressant exposure (El‐Mallakh et al., 2015; Frye et al., 2015; Ghaemi et al., 2010). Similarly, bipolar individuals who are treated for a depressive episode with an antidepressant (added to one or more mood stabilizers) are more likely to develop a chronic irritable dysphoric state (ACID for antidepressant‐associated chronic irritable dysphoria) than those treated without an antidepressant after they have recovered from the index depressive episodes (El‐Mallakh & Karippot, 2005; El‐Mallakh et al., 2008). Similar patterns may also occur in adolescents (Biederman et al., 2000; Hogg et al., 2016; Park et al., 2014) that may be associated with increased suicidal ideation (Baumer et al., 2006). Collectively, these findings suggest that an occult diagnosis of BD may play a role in poorer outcome with antidepressants in young people.

Additionally, young people may also have an overrepresentation of the short form of the SERT. The SERT protein is the target of serotonin reuptake inhibitors. It is coded for by the SoLute Carrier family 6 neurotransmitter transporter, serotonin, and member 4 gene (*SLC6A4*). Several genetic variants, the most common of which is a deletion of 44 base pairs in the promoter region (“s” or short form), result in a reduction of the number of serotonin reuptake pumps expressed in the synapse to about 50% of the individuals with the insertion of the 44 base pairs (“l” or long form) (Lesch et al., 1996; Margoob & Mushtaq, 2011). In understanding the interaction of this genetic variant with antidepressant medications, it is important to remember that a typical SRI must block roughly 80% of SERT proteins to achieve antidepressant effect (Meyer et al., 2004) so that individuals with the short form have similar biology to individuals receiving an SERT‐blocking SRI.

Adverse effects (AEs), severe AEs, or discontinuation due to AEs are more common in pediatric subjects receiving antidepressants than in adults (Locher et al., 2017). These very same problems are also more common in adults with the short form of SERT compared to those with the long form (Luddington et al., 2009). Additionally, the extent of benefit from antidepressants is reduced in children and adolescents compared to in adults (Locher et al., 2017; Walkup, 2017). Again, this is a characteristic that is seen in subjects who possess the short form of the SERT (Luddington et al., 2009). Children or adolescents who develop depression, particularly in the setting of childhood adversity, are more likely to have the short form than children without depression (Caspi et al., 2003; Haberstick et al., 2016; Talati et al., 2015).

Having the “s” allele is associated with an increased risk for suicide attempt or violent suicidal behaviors in adults (OR  =  1.44, CI 1.17–1.78, *p*  =   .0007) (Fanelli & Serretti, 2019; Lin & Tsai, 2004). Specific association with the s allele has been implicated in young people although adequate data are lacking. For example, treatment of depressed juveniles (7– 18 years) with citalopram resulted in more suicidal ideation in young people with two copies of the “s” allele compared to heterozygotes and long form homozygotes (Kronenberg et al., 2007).

More recently, several genome‐wide association studies (GWASs) have found no association between the short form and increased risk for depression, suicide, or a clear gene x environment interaction (Border et al., 2019; Culverhouse et al., 2018). These large studies have led researchers to discard the extensive literature regarding the short form, depression, and antidepressant response (Duncan et al., 2019). However, the short form has also been associated with *reduced* risk for depression compared to the long form in the setting of a positive environment (Bogdan et al., 2014; Kaufman et al., 2004; Li et al., 2013; Little et al., 2019). This reproducible finding suggests that in the absence of adversity, the “s” allele may be associated with an increase in fitness. These studies may help explain the relatively high prevalence of the allele in populations, ranging from a low of 0.23 in Africa to 0.43 in the United Kingdom and European Americans, to 0.7 in East Asia (Gelernter et al., 1999). Furthermore, these studies provide an explanation of why GWASs could not find an effect of *SLC6A4* (Border et al., 2019; Culverhouse et al., 2018); as the short form appears to be associated with *both* a reduced risk of depression (in the setting of positive environments) and an increased risk of depression (in the setting of significant adversity), neither effect would be visible in a GWAS analysis.(Cantor et al., 2010)

### 4.2 Lithium

Suicide is a major cause of mortality in affective disorders and notably, most suicide decedents suffered from a mood disorder (Barraclough et al., 1974; Cavanagh et al., 2003; Leahy et al., 2020). In patients with BD, overall mortality is two to three times that of the general population (Müller‐Oerlinghausen et al., 1992). In patients with BD who require hospitalization, the rate of suicide has been reported as 29 times greater without lithium maintenance (Nilsson, 1999). Fortunately, treatment with lithium appears to reduce that risk, even reducing mortality to levels comparable to the general population in some studies (Leahy et al., 2020). However, most studies show that even with the introduction of lithium, the 5‐year mortality and prognostic outlook of patients with BD remains elevated (Licht et al., 2008; Vestergaard & Aagaard, 1991).

One case‐control study found higher rates of psychopharmacologic treatment and lithium treatment in demographically matched controls with affective disorders who did not die of suicide compared to patients who completed suicide (Modestin & Schwarzenbach, 1992). A more recent case‐control study by Coryell and colleagues found that lithium was prescribed at similar rates to both patients who died by suicide and those who did not (Coryell et al., 2001).

A large systematic review and meta‐analysis including 6674 participants across 48 RCTs showed that in all mood disorders, lithium outperformed placebo with respect to number of suicides (OR 0.13, 95% CI 0.03–0.66) and mortality from any cause (OR 0.38, 95% CI 0.15–0.95) (Cipriani et al., 2013). When the same study examined unipolar depression alone, similar results were seen (risk of suicide OR 0.36, 95% CI 0.13–0.98, and overall mortality OR 0.13, 95%CI 0.02–0.76). Numerous other studies including meta‐analyses and RCTs have shown similar effects (Baldessarini et al., 2006; Coppen & Farmer, 1998; Lauterbach et al., 2008; Müller‐Oerlinghausen et al., 1992; Thies‐Flechtner et al., 1996; Tondo & Baldessarini, 2000; Tondo et al., 2001). The most recent meta‐review as of this writing examined 16 systematic reviews and found a consistent antisuicidal effect over 40 years of studies (Smith & Cipriani, 2017). According to the findings in this meta‐review, lithium is an underutilized treatment with considerable benefits in patients with affective disorders.

#### 4.2.1 Time course of effectiveness: Early effects

Although medications for mood disorders are often slow to achieve their therapeutic effect, the decrease in suicide rate appears to occur rapidly (within a month) after lithium initiation (Tsai et al., 2016). The hazard ratio of suicide‐related events (HR 0.10, 95% CI 0.06–0.15), completed suicide (no suicide death recorded in lithium condition), and all‐cause mortality (HR 0.03, 95% CI 0.03–0.05) were significantly lower compared to untreated patients. It is also interesting to note that the reduction in suicide associated with lithium appears to occur independent of the effect on mood in patients with recurrent affective disorders (Ahrens & Müller‐Oerlinghausen, 2001). Therefore, it may have a role in patients who do not find it helpful for their affective symptoms.

#### 4.2.2 Long‐term effects and after lithium discontinuation

The literature suggests that lithium prophylaxis requires lifelong adherence to be effective for persistence of reduction suicide risk. Numerous studies have found that cessation of lithium treatment, even after an extensive course, leads to the return of pretreatment levels of suicide risk. Kessing et al. examined patients in Denmark who purchased lithium from 1995 to 1999, comparing them to the general population, who did not purchase lithium and so may be assumed to have no psychiatric illness (Kessing et al., 2005). Although purchasing lithium at some point was associated with a higher suicide rate compared to the general population, patients who purchased lithium at least twice, implying continued treatment, had a reduced rate of suicide compared to purchasing lithium only once (OR 0.44, 95% CI 0.28–0.70). Increased purchases were associated with further reductions (Kessing et al., 2005). Nilsson and colleagues showed a 29‐fold increase in mortality off lithium and sevenfold increase in mortality on lithium (compared to the general population) in a high‐risk population with a previous psychiatric hospitalization and 1 year of lithium maintenance (Nilsson, 1999).

Maintenance treatment with lithium does not appear to confer any long‐term benefit after its cessation. For example, Bocchetta and colleagues found a five‐ to sixfold reduction in suicide attempts during lithium treatment in a patient population with previous suicide attempts, whereas the rates before initiation and after cessation were similar and high (Bocchetta et al., 1998). There is some evidence that the period immediately after lithium discontinuation represents a critical period of elevated suicide risk in patients with BD (Tondo et al., 1998). Clinically, this recommends closer monitoring when a patient's lithium treatment must be stopped, though the effect may be an artifact of the circumstances that led to medication nonadherence.

#### 4.2.3 Relative efficacy of lithium

In contrast to the strong evidence for lithium compared to placebo, the evidence comparing lithium to other medications is more mixed. Several studies suggest that treatment with other mood stabilizing agents yielded similar outcomes to lithium. For example, the rapid reduction in suicidal events compared to untreated patients, noted with lithium, was also noted in patients treated with divalproex (HR 0.14, 95% CI 0.11–0.19) and carbamazepine (HR 0.10, 0.07–0.16) (Tsai et al., 2016). Also, a naturalistic study of 140 patients with BD being treated for 6 months or more at a private practice showed that lithium, carbamazepine, and valproate were all associated with lower rates of nonlethal suicidal acts compared to patients that went off medication (χ^2 ^= 4.05, *p* = .04), but there were no significant differences seen within groups while they adhered to medication (χ^2 ^= 0.14, *p* = .93) (Yerevanian et al., 2003). A retrospective review of 405 veterans with BD followed for 3 years found that lithium, divalproex, and carbamazepine monotherapies were associated with similar rates of nonlethal suicidal acts, and that rates after discontinuation of any of these agents were significantly elevated, 16‐fold higher rate of nonlethal suicidal acts compared to the period during treatment (Yerevanian et al., 2007). Lithium did have the lowest overall rate of suicidal behaviors, but it does support the use of divalproex or carbamazepine in patients who cannot tolerate lithium. More recently, a study of patients with BD in Denmark showed that both lithium and valproate purchases were associated with lower suicide rates compared to periods where patients did not purchase these medications. Lithium was associated with a lower rate than valproate and switching or augmenting with lithium was associated with a significantly reduced rate of suicide in the population initially treated with valproate (rate ratio = 0.27, 95% CI 0.20–0.40) (Søndergård et al., 2008). When a large population of VA patients initiating lithium (*n* = 21) or valproate (*n* = 194) was examined retrospectively, there were no significant differences between suicide deaths between treatment groups in the first year (conditional odds ratio 0.86, 95% CI 0.82–1.81, *p* = .32) (Smith et al., 2014).

Where differential effects are found among treatments, they tended to favor lithium. One retrospective cohort study in California and Washington showed that suicide rates were 2.7 times higher in the patients treated with divalproex compared to lithium (Goodwin et al., 2003). Similarly, Ahern and colleagues found that, in a retrospective study of 1,306 veterans with BD, the lowest percentage of suicide attempts (15%) was in the group treated with lithium (Ahearn et al., 2013). When accounting for total months of exposure, the group treated with both valproic acid and lithium combined had the lowest overall suicide rate compared to monotherapy and other combination therapies. Lithium outperformed valproic acid alone and atypical antipsychotics (Ahearn et al., 2013). In a cohort of 826 patients with BD who attempted suicide previously and were then followed prospectively, lithium was associated with both decreased suicide mortality and decreased all‐cause mortality, whereas valproic acid, benzodiazepines, and antidepressants were all associated with an increased risk of attempted suicide (Toffol et al., 2015).

### 4.3 Applicable patient populations

Within psychiatry, lithium is most often considered for the treatment of BD due to its classification as a mood stabilizer. Although the literature clearly supports this application, it is worth noting that the benefits of lithium treatment extend to several patient populations. As noted above, lithium treatment has been associated with reduction in suicidal behaviors in patients with major depressive disorder, where it is generally used as an augmenting agent (Crossley & Bauer, 2007; Undurraga et al., 2019). The largest recent meta‐analysis that looked specifically at unipolar depression found lithium to be associated with an 85% reduction in suicide attempt and completions (Guzzetta et al., 2007). Although it has not been studied as extensively, there is some preliminary evidence for efficacy of lithium treatment in reducing suicidality among patients with Post‐Traumatic Stress Disorder (Gupta & Knapp, 2013) and Huntington's disease (Raja et al., 2013; Serafini et al., 2016). More research is needed to determine whether these findings are reproduced in prospective randomized studies. An additional surprising finding is that communities with higher levels of lithium in drinking water tend to have lower rates of suicide, homicide, and lower overall mortality (Barjasteh‐Askari et al., 2020; Giotakos et al., 2015; Ohgami et al., 2009), suggesting that submood‐stabilizing levels may still have an effect on suicide. Although the mechanism of lithium's antisuicide effect remains unclear, there is some evidence that it may extend to impulsive behaviors other than suicide attempts. A longitudinal cohort study in the United Kingdom showed lower rates of self‐harm in patients prescribed lithium compared to divalproex, olanzapine, or quetiapine (Hayes et al., 2016). Surprisingly, unintentional injury rates were also lower in this population.

Lithium's antisuicide effect appears to generalize to many populations but is unlikely to be universally useful. For example, in a case‐control study of patients with schizophrenia or schizoaffective disorder who died by suicide within 5 years of being diagnosed, there was no observed association between lithium and suicide prevention (Reutfors et al., 2013).

#### 4.3.1 Conclusions regarding lithium

Among populations that are high risk for suicide, the evidence clearly recommends that we consider lithium therapy; particularly in patients with a mood disorder, although possibly in people with other conditions as well. Limitations to its wider use include concerns about toxicity, need for close monitoring, concerns regarding thyroid and renal function, and possible interactions with other medications.

### 4.4 Clozapine

In addition to the high levels of persistent disability associated with schizophrenia, the illness is also attended by excess mortality that, according to a meta‐analysis of 11 studies, decreases life expectancy by 14.5 years (Hjorthøj et al., 2017). Suicide accounts for much of the excess mortality and occurs at a rate eightfold that of the general population (Harris & Barraclough, 1997). Despite often having adverse metabolic effects, antipsychotic treatment appears to substantially decrease overall mortality (Taipale et al., 2020). Evidence has emerged that shows that treatment with clozapine results in lower risks for death than treatment with other antipsychotics and that it does so largely through its effects of suicidal behavior. This evidence derives from studies that fall into four design categories: cohort registry, mirror image, controlled trials without random treatment assignment, and RCT.

One cohort registry study determined risks for suicidal acts in a large sample of patients with schizophrenia who were taking clozapine and compared these risks to those of all people with schizophrenia from the same geographical area who had not been exposed to clozapine (Reid et al., 1998). Another calculated comparison risks from a review of studies that provided standard mortality ratios for individuals with schizophrenia identified in clinical settings (Munro et al., 1999). Reid et al. reported a suicide rate of 12.7 per 100,000 patients/year among those taking clozapine in contrast to a rate of 63.1 among all other patients with schizophrenia or schizoaffective disorder (Reid et al., 1998). Among 12,760 patients who received clozapine over a 7‐year period in the Munro et al. series, the risk for suicide was five times that expected for the U.K. population, far less than the 20‐fold excess of suicide deaths predicted by a review of studies on mortality among individuals with schizophrenia in general (Munro et al., 1999).

Other cohort studies have compared patients with schizophrenia taking clozapine during a specified observation period to patients with schizophrenia under certain treatment conditions. Two of these compared suicide rates for those using specific antipsychotics to those on no antipsychotic (Haukka et al., 2008; Kiviniemi et al., 2013) and two others used an array of alternate antipsychotics for comparison (Reid et al., 1998; Ringbäck Weitoft et al., 2014). All found clozapine to be among the antipsychotics associated with the lowest rates of suicidal acts.

An exception is a report that identified 1415 inpatients in Veterans Administration facilities over a 3‐year period who were exposed to clozapine while hospitalized (Sernyak et al., 2001). Suicide rates among patients exposed to clozapine did not differ from unexposed inpatients with schizophrenia. The large sample size comprised a relative strength of this study. This was offset, however, by the fact that observation of clozapine exposure was limited to that which took place in inpatient settings.

Three mirror‐image studies, comparisons of suicidality while on clozapine treatment, compared to periods off clozapine, were consistent in finding lower rates of suicidal acts in the former condition (Meltzer & Okayli, 1995; Modestin et al., 2005; Walker et al., 1997). Meltzer et al. described 88 neuroleptic‐resistant patients with schizophrenia placed on clozapine and found that 17 (19.3%) had made attempts before clozapine was begun while only three (3.4%) made attempts during clozapine treatment (χ ^2 ^= 11.0, df = 1, *p* < .001) (Meltzer & Okayli, 1995). The authors did not specify that the periods of risk before and during clozapine treatment were equal. Modestin et al. presented rates of suicide attempts before clozapine treatment was instituted in a group of 94 inpatients and compared them to rates in an equal period of prospectively observed clozapine treatment. Proportions with suicide attempts were 26 (27.6%) and 3 (3.2%), respectively (χ^2 ^= 21.6, df = 1, *p* < .001) (Modestin et al., 2005).

Walker et al. used a different approach and linked a clozapine registry with National Death Index mortality data (Walker et al., 1997). Within 15,763 person‐years for individuals with past clozapine use, there were 33 suicides for an adjusted rate of 316 suicides/100,000 person‐years. The adjusted rate for current clozapine users was 39 for a rate ratio of 0.17 (95% CI 0.10–0.30) (Walker et al., 1997).

Spivak's group conducted an evaluation of the suicide item on the Hamilton Depression Scale (HAM‐D) in a 6‐month, open, nonrandomized comparison of depot haloperidol and clozapine (Spivak et al., 2003). Baseline and 6‐month mean values were 2.2 (1.1) and 0, respectively, for the group treated with clozapine (*p* = .01) but were 2.4 (1.1) and 1.1 (0.9), for the group treated with haloperidol. Notably, the reduction in HAM‐D suicide item ratings for the clozapine group correlated significantly with reductions in impulsivity (*r* = 0.75, *p* < .03) and aggression (*r* = 0.8, *p* < .03) scale scores. Reductions in impulsivity and aggression scale scores did not correlate significantly in the group treated with haloperidol (Spivak et al., 2003). Reductions in aggression with clozapine frequently occur independent of antipsychotic effect (El‐Mallakh & McKenzie, 2013). Thus, the mechanism by which clozapine might decrease risks for performing suicidal acts may operate through its effects on impulsivity and aggression rather than through its often‐cited superiority in global efficacy.

The most compelling evidence for the antisuicidal effects of clozapine derives from a multinational randomized, 2‐year study that compared clozapine to olanzapine (*n* = 490 per each group) (Meltzer et al., 2003). All patients were seen with the same frequency to control for the closer monitoring required for clozapine. Patients with treatment refractory schizophrenia randomized to clozapine had significantly fewer suicide attempts (34 vs. 55; *p* = .03) as well as fewer interventions needed to prevent suicide such as hospitalizations (82 vs. 107; *p* = .05) or other rescue interventions (118 vs. 155; *p* = .01), and fewer prescriptions for antidepressants (221 vs. 258; *p* = .01) (Meltzer et al., 2003). These researchers were mindful of the possible artifact produced by the necessarily high clinical oversight attending clozapine treatment and instituted the same visit schedule for the olanzapine group and for those receiving clozapine. The report, however, did not address the issue of whether clozapine's antisuicidal effect was accounted for overall symptom improvement.

Two important caveats must accompany any conclusions that the studies described above have demonstrated that clozapine has benefits specific to suicidal acts. First, clozapine has well‐established superiority over conventional antipsychotics in overall symptom control in patients with treatment resistant psychosis (Kane et al., 1988), and lower rates of suicidal acts may simply reflect better symptom control in general. Second, risks for blood dyscrasias unique to the use of clozapine require closer clinical monitoring and this, in itself, may have resulted in lower rates of suicidal behavior. The observations that treatment with other specific antipsychotics also lower risks for suicidal acts relative to the absence of antipsychotic treatment (Haukka et al., 2008; Ringbäck Weitoft et al., 2014; Taipale et al., 2020) comprise evidence of this possibility.

#### 4.4.1 Conclusions regarding Clozapine

In summary, studies using a variety of designs have shown, with near uniformity, that treatment with clozapine lowers risks for suicidal acts relative to no treatment or to treatment with most other antipsychotics. Reasons for this are unclear and antipsychotic treatment, in general, appears to reduce risks for suicide or suicide attempts. A possible rise of suicidality with clozapine withdrawal, as happens with lithium, has not been investigated. Whether the greater effectiveness of clozapine in symptom control accounts for part or all of its value as an antisuicidal agent is also unclear and is likely to be a fruitful area of future research.

### 4.5 Intravenous ketamine

Although other treatments outlined in this review may have demonstrated antisuicidal efficacy, their clinical utility is often limited by an initial titration period with a relatively delayed response. Ketamine, an *N*‐Methyl‐d‐aspartate (NMDA) receptor antagonist, has emerged as an attractive candidate in this aspect due to its quick onset as initially described in a study by Berman et al. in which a single ketamine infusion significantly improved depressive symptoms within 72 h (Berman et al., 2000). Numerous RCTs have subsequently evaluated the effect of a single dose of IV ketamine on depression, with most of the patients experiencing suicidal ideation. The overwhelming majority of these studies have used a similar dose and format, namely, ketamine dosed at 0.5 mg/kg and infused over 40 min. Several reviews of these data have found a significant antisuicide effect with IV ketamine in depressed patients.

Individual patient‐level data of 298 patients from 10 ketamine trials published from 2000 through 2016 were compiled by Wilkinson et al. (2018), from which 167 patients were identified as reporting baseline suicidal ideation. Using both clinician and patient self‐reports of suicidal ideation, these 167 individuals provided data on suicidal ideation at days 1, 2, 3, and 7 post the ketamine infusion. The researchers concluded that a single dose of IV ketamine rapidly reduced suicidal thoughts within 1 day and for up to 1 week, with a moderate to large effect size (Cohen's *d* = 0.85 on day 1 and *d*  = 0.61 on day 7) (Wilkinson et al., 2018). After controlling for improvement in severity of depressive symptoms, ketamine's effects on suicidal ideation remained significant. Thus, a specific antisuicidal effect of IV ketamine has been established from high‐quality RCTs.

A Cochrane review by Caddy et al. (Caddy et al., 2015) found “evidence that ketamine was more effective than midazolam in reducing suicidal ideation (mean difference—1.32, 95% CI −2.52 to −0.12; *p *= .03)” based on the single randomized control trial by Murrough and colleagues and two post‐hoc analyses of the same study (Murrough et al., 2013; Murrough et al., 2015; Price et al., 2014). The original trial was also included in the aforementioned systematic review by Wilkinson and colleagues (Wilkinson et al., 2018).

Ballard et al. took data from five RCTs of IV ketamine for unipolar or bipolar depression (total *n* = 128) and evaluated the overall impact on suicidality, exploring which factors might predict response (Ballard et al., 2018). Overall, both depression remitters and responders had significant antisuicidal response that was partly independent of the magnitude of depression improvement, with maximal effect 1‐day postinfusion. Individuals with chronic suicidality showed limited change.

Four additional RCTs published after 2016 specifically recruited patients with depression and suicidal ideation. Grunebaum et al. conducted a double‐blind randomized control trial of ketamine versus midazolam, with just 16 patients receiving a single infusion (Grunebaum et al., 2017). The seven patients receiving ketamine had a six‐point greater reduction in the Scale for Suicidal Ideation (SSI), in comparison to the nine patients receiving midazolam, but this was not statistically significant, likely due to small sample size (Grunebaum et al., 2017). Additional exploratory findings revealed statistically significant correlations between reduction in SSI score on day 1 and memory improvement (measured by improvement on the Selective Reminding Test) as well as postinfusion decreases in serum brain‐derived neurotrophic factor (BDNF) (Grunebaum et al., 2017).

The following year, the same group reported a similarly designed RCT involving a comparison of a single infusion of IV ketamine or midazolam for individuals with unipolar depression and clinically significant suicidal ideation, again using the SSI at 24 h postinfusion as the primary outcome (Grunebaum et al., 2018). On day 1, the 40 individuals who received ketamine had a 4.96‐point greater reduction in SSI compared to the 40 individuals who received midazolam, statistically significant with a medium effect size (Cohen's *d* = 0.75) (Grunebaum et al., 2018). Improvement in suicidality was maintained through the 6‐week study period, but patients were also receiving additional pharmacotherapy. Additional analyses suggested that ketamine's effects on depression and suicidal thoughts are at least partially independent (Grunebaum et al., 2018).

Ionescu et al. conducted an RCT of IV ketamine (0.5 mg/kg) versus IV saline in six separate infusions over a 3‐week period involving 26 outpatients with severe depression and chronic suicidal ideation, half of whom had not responded to ECT (Ionescu et al., 2019). Patients continued to receive other oral antidepressants in an uncontrolled fashion. No differences in suicidality were found between groups after the six infusions using the Columbia Suicide Scale; however, it is noteworthy that neither group had much improvement in depression symptoms. The authors concluded that consistent with some of their own previous research (Ionescu et al., 2016), the dose of ketamine may have been insufficient in severely and chronically ill patients.

Chen et al. conducted a double‐blind, randomized, three parallel‐group, placebo‐controlled trial of a single infusion (saline, ketamine 0.2 mg/kg, ketamine 0.5 mg/kg) in 71 Taiwanese patients with refractory depression. Using the suicide item on the HAM‐D and the Montgomery–Åsberg Depression Rating Scale (MADRS) scales, they found that only 0.5 mg/kg dose of ketamine was effective in reducing suicidal ideation and did so for up to 2 weeks postinfusion (Chen et al., 2019). They also evaluated the potential role of BDNF Val66Met polymorphism as a mediator of ketamine's antidepressant effects and found that presence of one or two valine alleles of BDNF was predictive of a positive response to ketamine (Chen et al., 2019).

High‐quality research in the form of RCTs may also be supplemented by open‐label and quasi‐experimental studies gathered from the search parameters. Some of these ancillary findings are worth noting. Several open‐label studies offer preliminary evidence that repeats infusions (e.g., six infusions over 12 days) can help sustain acute improvement in suicidality in treatment responders (Price et al., 2009; Sinyor et al., 2018; Vande Voort et al., 2016; Zhan et al., 2019; Zheng et al., 2018).

Single ketamine infusions have also been briefly evaluated in the treatment of other subtypes of depression. A randomized control trial of 654 Chinese women undergoing cesarean section suggested that prophylactic ketamine (0.5 mg/kg) versus placebo infusion may be protective against postpartum depression at 4 days and 6 weeks, with some impact on suicidality (3% of ketamine‐treated women being free of suicidal ideation by 6 weeks versus 7.7 % of placebo treated women) (Ma et al., 2019). An RCT of 42 Chinese patients with newly diagnosed cancer patients found that patients receiving ketamine (0.5 mg/kg) had a significant reduction in total score and of the suicide item on the MADRS on days 1 and 3, but not by day 7 (Fan et al., 2017).

#### 4.5.1 Conclusions regarding ketamine

In summary, controlled data from a variety of studies provide robust evidence of a specific, ant‐suicidal effect of IV ketamine in depressed individuals with significant suicidality. These effects are notable particularly for the rapidity of antisuicidal efficacy, and for suggesting that the benefit is in part independent of the global improvement in depression. However, the antisuicidal efficacy is relatively transient after a single infusion, typically lasting no longer than 1 week. It may be made more sustained with repeated infusions. Individuals with chronic suicidality are less likely to respond. Using ketamine to treat suicidality, like acute use of an emergency room or a brief hospitalization, may be a critical element in reducing depression mortality, but will need integration into a comprehensive approach to treatment of depression and suicidality. The antisuicidal efficacy of IV ketamine in other illnesses such as anxiety disorders remains to be established.

### 4.6 Intranasal esketamine

The S enantiomer of ketamine, esketamine, administered via an intranasal route has been developed by Janssen as a novel antidepressant, and approved by the US FDA on March 5, 2019 (Food & Drug Administration, 2019), as adjunct for treatment resistant depression. More recently, on August 3rd, 2020, esketamine was approved for use in patients with major depressive patients with acute suicidal ideation (Janssen Pharmaceuticals, 2020). Compared to the R‐ketamine enantiomer, esketamine has a two to threefold greater affinity for the NMDA receptor (Hirota et al., 1999). Similar to ketamine, esketamine is believed to produce a glutamate burst by inhibiting γ‐aminobutyric acid (GABA) interneurons that modulate glutamatergic neurotransmission (Duman et al., 2016). It appears to share IV ketamine's ability to rapidly improve depressive symptoms and reduce suicidal ideation and other depressive symptoms (De Berardis et al., 2018).

Several company‐sponsored studies have been conducted to determine the effects of esketamine on suicidality. A Phase 2 double‐blind, multicenter, placebo‐controlled study evaluated the efficacy and safety of intranasal esketamine in comparison to an intranasal placebo in patients with MDD and active suicidal ideation (Canuso et al., 2018). Sixty‐six participants were randomly assigned to two groups, one given intranasal esketamine (84 mg) and the other given placebo twice weekly for a span of 4 weeks in addition to comprehensive standard‐of‐care treatment, including hospitalization. Although there was a decrease in the suicide item on the suicide item on the MADRS in the esketamine group at the 4‐h time point (effect size = 0.67). Numerically, there was a greater improvement in the depressive symptoms in the esketamine group in comparison to the placebo group at all time points. The study is unique in that it recruited patients with MDD and active suicidal ideation with intent. The results demonstrated that intranasal esketamine can rapidly reduce depressive symptoms, which included suicidal ideation, in patients with an imminent risk for suicide. Limitations of the study included the small sample size, the lack of sample diversity limited to those in the United States, and the exclusion of patients with psychotic symptoms or BD (Canuso et al., 2018).

In a phase 3, double‐blind, multicenter study (named ASPIRE), 226 patients with MDD and active suicidal ideation with intent, and who were psychiatrically hospitalized, were randomly assigned to either intranasal esketamine (84 mg) or placebo nasal groups. Each group received treatment twice weekly for a span of 4 weeks while receiving comprehensive standard treatment of care, including a new or optimized antidepressant (Fu et al., 2020). Both esketamine and placebo groups showed improvement in MADRS score from baseline to 24 h after the first dose but the esketamine group showed a significantly greater improvement (*p* = .006). Unlike the phase 2 trial, this study recruited patients globally. In both the esketamine group and the placebo group, there was a drop in the severity of their suicidality, as examined at 24 h by CGI‐S (Fu et al., 2020). Contrasting to Canuso's phase 2 study, there was no statistical difference between the treatment groups at the end of the trial. The hospitalization on a psychiatric unit and allowing patients to use benzodiazepines may have influenced the rapid drop in suicide severity in both groups.  Limitations of the study include methodological challenges required for ensuring safety when studying suicidal patients, such as separating the benefits from esketamine and that of the hospitalization and care provided by the healthcare professionals.

As of September 4, 2018, there were a total of six deaths of which three were suicides among the 3549 subjects who had participated in the esketamine studies (Kim & Chen, 2019). All deaths occurred in subjects randomized to esketamine, but none were determined to be due to esketamine, either directly or indirectly. This conclusion was also reached regarding the three suicides which all occurred in subjects that were receiving or had received esketamine. There were no deaths, and no suicides in the placebo group (Kim & Chen, 2019).

#### 4.6.1 Potential mechanisms of action

Mood disorders and suicidal acts are believed to be the result of deeply intertwined dysfunctions of cognition and neurobiology. It is believed that ketamine and esketamine target many of the impaired neural connections, leading to their antidepressant and possible antisuicidal effects. Specifically, ketamine/esketamine may alter prefrontal cortical (PFC) function. The PFC plays a significant role in the cognitive deficits displayed by depressed and suicidal patients. Such patients display “attentional bias”—a tendency to pay more attention to environmental cues related to their suffering. Depressed patients who have attempted suicide also performed worse in memory, attention, and working memory tasks, indicating executive dysfunction. Impulsivity and aggression, both of which have been related to a higher number and lethality of suicide attempts, have been associated with abnormal volumes across fronto‐temporal‐limbic regions of the brain (involved in cognitive control of emotions and behavioral planning) (De Berardis et al., 2020; Ghosal et al., 2017). GABA and glutamate are disrupted in many ways in the PFC of depressed patients (De Berardis et al., 2020), and gene expression related to these neurotransmitters is altered in patients who died from (Zhao et al., 2018). In animal models, ketamine infusion into the infralimbic PFC appears to mediate its antidepressant action (Fuchikami et al., 2015).

There are a few prevailing theories on how ketamine stabilizes the glutamate‐GABA imbalance in the PFC. Disinhibition theory claims that ketamine blocks NMDA receptors on tonic firing GABA interneurons, causing a glutamate surge from pyramidal neurons in the PFC. Consequently, the glutamate activates alpha‐amino‐3‐hydroxy‐5‐methyl‐4‐isoxazolepropionic acid (AMPA) receptors, which triggers two main elements of intracellular transductional pathways, namely—mammalian target of rapamycin (mTOR) and BDNF (Duman et al., 2016). Another hypothesis by Autry et al. proposes that ketamine directly inhibits postsynaptic NMDA receptors on pyramidal neurons, preventing phosphorylation of eukaryotic elongation factor 2 (eEF2), resulting in rapid BDNF translation (Autry et al., 2011). A third theory includes ketamine's action on extra‐synaptic NMDA receptors, which are activated by ambient glutamate, which causes disinhibition of mTOR. All theories, however, agree on mTOR and BDNF being the main targets for ketamine's action (Lengvenyte et al., 2019). All of these theories have important practical considerations. For example, if the disinhibition theory is correct, coadministration of esketamine with a benzodiazepine may alter the response because the effect on the GABA interneurons would be related to the interaction of esketamine and the benzodiazepine. Such an interaction may have been the reason for the failure of the phase 3 study cited above to have separated from placebo (Fu et al., 2020).

#### 4.6.2 Summary of esketamine

Esketamine is the only medication approved by the US FDA for use in patients with active suicidal ideation. However, it is not approved for suicidal ideation. Patients in the pivotal studies did experience a dramatic reduction in suicidal ideation, but so did the control group, and the difference was significant only within 4 h of administration. It is likely that the antisuicide effect is bigger than measured in these pivotal trials. However, it is important to be cautious because completed suicide occurred in three patients who took esketamine, but none of the patients who were given placebo. Clearly, additional research is needed to understand the clinical effects of this intervention.

### 4.7 Oral and intramuscular ketamine

The treatment of depression with intranasal and IV esketamine and ketamine dominates the literature, but there exists a small number of studies looking at oral or intramuscular (IM) ketamine and the role it can play in depression and suicide. The bioavailability of oral ketamine is very poor with less than 25% of administered oral dose reaching the venous circulation (Chong et al., 2009; Clements et al., 1982). Furthermore, there appears to be significant variability between studies (17–45%) (Andrade, 2019).

Most of the information regarding oral ketamine derives from case reports that do not use any standardized dosage paradigm and demonstrate incredible variability in response (Dadiomov & Lee, 2019). However, in randomized, controlled trails, oral ketamine appears to display antidepressive effects (Arabzadeh et al., 2018; Domany et al., 2019) but the effect size appears to be significantly smaller than either IV ketamine or intranasal esketamine (McIntyre et al., 2020) and the onset may be delayed (Rosenblat et al., 2019). An antisuicide effect as a consequence of improvement in depression or independent of that effect has not been adequately examined but is alluded to in some case reports (e.g., De Gioannis & De Leo, 2014)

Similarly, IM ketamine has also been studied in case reports and small trials (Chilukuri et al., 2014; Loo et al., 2016). As with oral ketamine, positive effects on suicide ideation are exclusively anecdotal (e.g., Bigman et al., 2017; Harihar et al., 2013).

### 4.8 Electroconvulsive therapy

ECT is generally believed to be an appropriate treatment for highly suicidal patients. However, the quality of data on which this belief is based is somewhat low.

Avery and Winokur studied 519 patients hospitalized for depression 6 months following discharge (Avery & Winokur, 1978). They divided patients into treatment groups including ECT, antidepressant treatment, adequate antidepressant treatment, and none of the above. The patients receiving ECT had significantly fewer suicide attempts during the follow‐up period; however, this group of patients also had fewer suicide attempts by history prior to hospitalization.

Prudic and Sackheim (1999) and Kellner et al. (2005) demonstrated a short‐term decrease in suicidality in depressed patients receiving ECT. The strength of the effect was not compared to similar effects from other treatments. Prudic and Sackheim also reviewed the literature on long‐term effects, supporting a decreased all‐cause mortality in patients receiving ECT (Avery & Winokur, 1976; Tsuang et al., 1979). However, Milstein et al. did not find a decrease in suicide specifically in patients receiving ECT compared to other treatments (Milstein et al., 1986).

O'Leary et al. analyzed suicide rates in persons with affective disorders by treatment era (O'Leary et al., 2001). They found a significant drop in suicide during the era in which ECT was introduced (1940–1959) and a further drop in the era in which antidepressants were introduced (1960–1995). The baseline period was 1900–1939. These historical data are hard to interpret because the specific treatments for individual patients are not enumerated and because there is no control for nonspecific treatment factors or patient characteristics.

Sharma reviewed the literature and identified methodologic limitations of existing studies on ECT as a prevention for suicide (Sharma, 2001). He noted that the studies were mostly small samples, studied retrospectively, with nonrandom assignment. Often pertinent demographic and clinical variables were not provided, and contemporaneous control groups with similar follow‐up periods were often not available.

Mehlum et al. (2006), on behalf of the Norwegian Health Service, performed a systematic review of the literature to assess the effects of interventions in preventing suicide. No methodologically acceptable study on the preventive effect of ECT on suicide was found.

In a meta‐analysis of published studies of patients with TRD, Bergfeld et al. summarized the effect of multiple TRD treatments (including ECT, DBS, capsulotomy, vagal nerve stimulation, epidural cortical stimulation, CBT, esketamine, and treatment as usual) on completed suicide (Bergfeld et al., 2018). The data set consisted of 28 studies including a total of 1720 patients followed for an average of 149 weeks. Five of these studies were testing the effects of ECT, including 187 patients. No difference between treatments on risk for suicide could be confirmed.

#### 4.8.1 Conclusions regarding ECT

It is reasonable to conclude that ECT has a significant effect in reducing suicidal ideation following acute treatment. We cannot conclude that this effect is greater than that of antidepressants. Long‐term preventive effects of ECT on suicide cannot be confirmed. The ideal study has not yet been carried out. It would require random assignment of patients to ECT versus an antidepressant treatment and follow‐up over at least 1–2 years. As suicide is a rare event during a given year, even in patients with mood disorders, it would require substantial attention to determine adequate sample size. Nonspecific treatment effects, including the effect of follow‐up itself, would need to be controlled.

### 4.9 Summary

Suicide is a major public health problem globally. The majority of patients who die by suicide have a significant psychiatric illness. Somatic psychiatric treatments clearly reduce symptomatic and functional morbidity, but their effect on mortality is less clear. Only two medications, lithium and clozapine, have been shown to reduce suicide risk. The antisuicide effect has only been demonstrated in specific diagnostic groups: BD for lithium and schizophrenia for clozapine. The utility of these pharmaceuticals in other diagnoses is unknown. Furthermore, lithium discontinuation is associated with a markedly increased suicide risk. It is unknown if clozapine discontinuation may have a similar rebound effect. Ketamine, esketamine, and ECT may have a transient antisuicide effect. The duration of this effect is not fully clear, but which appears to be briefest with intranasal esketamine, minimally longer with IV ketamine, and longer with ECT. Ketamine and esketamine eventually may be useful for emergent management of suicidality. The effect of antidepressants is not at all clear. There appears to be direct evidence for antidepressants increasing suicidal ideation and the risk for suicide over the short term in young people, but indirect (low quality) evidence that antidepressants reduce suicide risk over the long term.

### PEER REVIEW

The peer review history for this article is available at https://publons.com/publon/10.1002/brb3.2381

## Data Availability

There are no data for review for this manuscript other than the papers reviewed.
